# Supplementary material for: Voltage-Gated Sodium Channels as Potential Biomarkers and Therapeutic Targets for Epithelial Ovarian Cancer
Source: Cancers (Basel). 2021 Oct 29;13(21):5437. doi: 10.3390/cancers13215437 (PMC8582439; doi:10.3390/cancers13215437)
Supplement: Supplementary file 1 [file cancers-13-05437-s001.zip › cancers-1424171-supplementary - new.pdf]

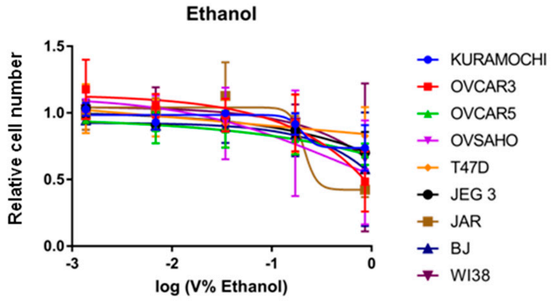


**Figure S1.** Dose response curves for ethanol control. Cells were treated with isovolumetric percentages of ethanol compared to volumes of local anesthetics utilized in the dose response experiments.

**Table S1. IC50[a] values for local anesthetics by cell line**

| | | Local Anesthetic | | |
|---|---|---|---|---|
| | | **Lidocaine** | **Bupivicaine** | **Procaine** |
| **Tissue type** | **Cell Line** | **IC50 (mM)** | **IC50 (mM)** | **IC50 (mM)** |
| Ovarian cancer | KURAMOCHI | 7.56 | 2.16 | NR[b] |
| Ovarian cancer | OVCAR3 | 5.56 | 1.35 | 24.44 |
| Ovarian cancer | OVCAR5 | 6.01 | NR | 10.34 |
| Ovarian cancer | OVSAHO | 6.59 | 0.81 | 3.12 |
| Breast cancer | T47D | 5.56 | NR | 3.24 |
| Choriocarcinoma | JEG 3 | 4.18 | 3.14 | 3.53 |
| Choriocarcinoma | JAR | 3.50 | 0.58 | 3.05 |
| Fibroblast | BJ | 11.38 | 0.96 | 8.04 |
| Fibroblast | WI38 | 5.44 | 3.16 | 10.71 |

[a] IC50 – Half maximal inhibitory concentration

[b]NR – not reached

[c] – IC50 values for benzocaine not reached
